# To metabolomics and beyond: a technological portfolio to investigate cancer metabolism

**DOI:** 10.1038/s41392-023-01380-0

**Published:** 2023-03-22

**Authors:** Federica Danzi, Raffaella Pacchiana, Andrea Mafficini, Maria T. Scupoli, Aldo Scarpa, Massimo Donadelli, Alessandra Fiore

**Affiliations:** 1 Department of Neurosciences, Biomedicine and Movement Sciences, Section of Biochemistry, University of Verona, Verona, Italy; 2 Department of Diagnostics and Public Health, University of Verona, Verona, Italy; 3 Department of Neurosciences, Biomedicine and Movement Sciences, Biology and Genetics Section, University of Verona, Verona, Italy; 4 ARC-NET Research Centre, University and Hospital Trust of Verona, Verona, Italy

**Keywords:** Biochemistry, Cancer metabolism

## Abstract

Tumour cells have exquisite flexibility in reprogramming their metabolism in order to support tumour initiation, progression, metastasis and resistance to therapies. These reprogrammed activities include a complete rewiring of the bioenergetic, biosynthetic and redox status to sustain the increased energetic demand of the cells. Over the last decades, the cancer metabolism field has seen an explosion of new biochemical technologies giving more tools than ever before to navigate this complexity. Within a cell or a tissue, the metabolites constitute the direct signature of the molecular phenotype and thus their profiling has concrete clinical applications in oncology. Metabolomics and fluxomics, are key technological approaches that mainly revolutionized the field enabling researchers to have both a qualitative and mechanistic model of the biochemical activities in cancer. Furthermore, the upgrade from bulk to single-cell analysis technologies provided unprecedented opportunity to investigate cancer biology at cellular resolution allowing an in depth quantitative analysis of complex and heterogenous diseases. More recently, the advent of functional genomic screening allowed the identification of molecular pathways, cellular processes, biomarkers and novel therapeutic targets that in concert with other technologies allow patient stratification and identification of new treatment regimens. This review is intended to be a guide for researchers to cancer metabolism, highlighting current and emerging technologies, emphasizing advantages, disadvantages and applications with the potential of leading the development of innovative anti-cancer therapies.

## Introduction

**Fig. 1:**
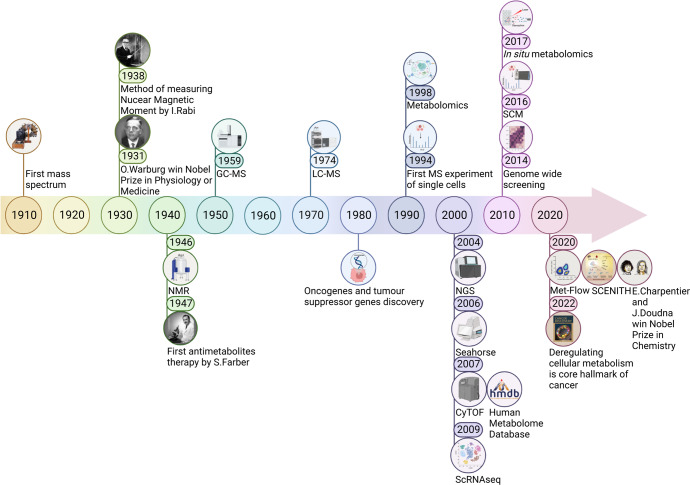
Timeline of the milestone events for cancer metabolism. The first mass spectrum of a molecule was measured by Joseph J. Thomson in 1910. In 1931 Otto H. Warburg won the Nobel Prize in Physiology or Medicine for characterizing the respiratory enzyme. In 1938 Isidor I. Rabi detected the Nuclear magnetic resonance (NMR) for the first time in a beam of lithium chloride thus developing the methodology and further expanded in 1946 by Felix Bloch and Edward M. Purcell for use on liquids and solids. Gas chromatography (GC)-MS was described in 1959 and Liquid chromatography (LC)-MS was introduced in 1974. The discovery of oncogenes and tumour suppressor genes dates back to the 1980s. In 1994 Tsutomu Nomizu and colleagues realized the first single cells MS experiment, while in 1998 Steven Oliver firstly introduced the concept of metabolomics. 2004 is the year of Next-generation sequencing (NGS), 2006 of the Seahorse real-time cell metabolic analyser, 2007 of the first prototype of Cytometry by Time-of-flight (CyTOF) and of the first version of The Human metabolome database (HMDB). In 2009 there was the development of Single-cell RNA sequencing (scRNAseq), in 2016 of Single-cell metabolomics (SCM) and in 2017 of the In situ metabolomics. The first genome-wide functional screening was performed in 2014 and in 2020 Emmanuelle Charpentier and Jennifer Doudna were awarded the Nobel Prize in Chemistry for discovering the CRISPR/Cas9 system. In 2020 the flow-cytometry-based technologies Met-flow and SCENITH have been proposed. In 2022 the deregulation of cellular metabolism was eventually recognized core hallmark of cancer by Douglas Hanahan. This figure was created with Biorender.com

Tumour cells rewire their metabolism in order to drive and promote the growth and spreading of cancer cells.^1^ This metabolic rewiring causes a unique metabolic phenotype, whose features can be exploited for biomarkers discovery, disease prediction, cancer diagnosis, patient stratifications and personalized therapies.^2–4^ Metabolic alterations of malignant cells have long been a central focus in the field of cancer research and currently the deregulation of cellular metabolism is a recognized core hallmark of cancer in modern medicine^5,6^ (Fig. 1). The field of cancer metabolism put down roots in the 1920s with the pioneered studies of Otto H. Warburg who hypothesized, for the first time, that cancer, compared to a normal tissue, utilizes enormous amounts of glucose to generate lactic acid, even in normoxia or in the presence of non-hypoxic conditions. This process was named the “Warburg effect” and its molecular explanation completely prioritized the following years of cancer research.^7–10^ During the 1980s, the search for abnormal metabolic pathways that can be therapeutically exploited drastically accelerated, leading to the discovery of oncogenes and tumour suppressor genes that are the basis for alternative metabolic pathways in cancer. The uncontrolled cell proliferation, increased cell survival, cell differentiation, immune surveillance, aberrant angiogenesis, and many other metabolic events linked to cancer are strictly associated with the oncogenic activities of mutant KRAS, mutant p53, activation of mTOR signalling and of the transcription factor c-MYC, among the others.^11–19^ Human cancers are characterized by an extreme context-dependent plasticity and diversity; indeed, they develop multiple metabolic alterations, as part of a multistep process, to fulfil the bioenergetic, biosynthetic, and reduction–oxidation (redox) demands of malignant cells.^20^ The most common metabolic alterations include: (i) cell survival and anabolic growth during nutrient deprivation, which is sustained via the acquisition of nutrients from the extracellular space and their conversion into macromolecules through the core metabolic pathways, such as glycolysis, Pentose phosphate pathway (PPP) and Tricarboxylic acid cycle (TCA).^21–24^ The increased uptake of amino acids and glucose, with consequent release of lactate and protons to the tumour microenvironment, is further enhanced via more mechanical processes, such as phagocytosis, macro-pinocytosis and entosis;^25–31^ (ii) increased acquisition and utilization of nitrogen, building block for the de novo synthesis of a variety of biological compounds including nucleotides, amino acids, polyamines, hexosamines, Glutathione (GSH), porphyrins, ammonia, creatine, nitric oxide;^32,33^ (iii) extensive metabolic enzyme-mediated and metabolite-mediated modulation of gene expression including methylation, acetylation, phosphorylation, ubiquitination of histones and succinylation; (iv) balanced Reactive oxygen species (ROS) by fine-tuning redox systems: ROS cause DNA damage, contributing to the appearance of oncogenic mutations, and acting as pro-growth signals thus sustaining tumour initiation and progression as well as angiogenesis and metastasis. Therefore, a tumour generally depends more than a normal tissue upon the antioxidants, including PPP-derived Nicotinamide adenine dinucleotide phosphate (NADPH), GSH, thioredoxin (TXN), antioxidant enzymes [i.e., mitochondrial and cytosolic peroxiredoxins (PRXs), glutathione peroxidase (GPXs), catalase and Superoxide dismutase (SOD)] and their transcriptional master regulators, as the Nuclear factor erythroid 2-related factor 2 (NRF2)^34–43^; (v) accumulation of oncometabolites, such as succinate, fumarate, and 2-hydroxyglutarate (2HG), which have higher concentration in malignant cells as a consequence of respectively loss-of-function mutations within the genes encoding succinate dehydrogenase (SDH), Fumarate hydratase (FH) or gain-of-function mutations in the Isocitrate dehydrogenase 1 or 2 (IDH1 or IDH2) genes. The increased level of any of these oncometabolites has been associated with a pro-tumorigenic effect in certain tissues.^44–48^

The idea of targeting metabolism is not a new concept; the first antimetabolites therapy dates back to 1947 when Dr. Sidney Farber, the father of the modern chemotherapy, first discovered that aminopterin administration to children affected by Acute lymphoblastic leukaemia (ALL) may arrest tumour progression (Fig. 1). Aminopterin is a folate analogue that inhibits the de novo nucleotide biosynthesis by blocking the one-carbon transfer reaction and its clinical success led to the development of an entirely new class of drugs.^49–55^ The introduction of antimetabolites probably constitutes one of the biggest achievements in the field of cancer metabolism: Methotrexate (MTX, amethopterin) a synthetic and less toxic derivative of aminopterin, the uracil analogue 5-fluorouracil (5-FU) and the nucleoside analogues gemcitabine and cytarabine are all notable examples of widely used antimetabolites employed in a variety of cancer settings over the years.^56–58^ Despite these successes, until now only a few metabolic liabilities have been translated into effective therapies. Toxicity versus normal cells and the intrinsic plasticity of cancer, which allow cells to activate alternative pathways in response to metabolic drugs, make particularly challenging targeting metabolism in these cells thus highlighting the urgent need to further investigate cancer biology with the final goals of the identification of successful screening and personalized therapeutic strategies to improve patient care.

Despite the massive effort, the metabolic reprogramming of cancer cells remains far from being fully characterized because of its complexity and due to technical limitations. Currently, we recognize the multidisciplinary of cancer metabolism that sits at the interface between numerous technical-scientific disciplines and approaches, in particular biochemistry, immunology, genetics and microscopy. This knowledge brought tremendous technological advancement in metabolites characterization and quantification, functional analysis of metabolic activities, innovative single-cell and genetic approaches that have given researchers specific and potent tools to untangle the complexity of cancer metabolism. Selecting the best method or the combination of methods that is more appropriate for a given study depends on equipment availability, budget, and scientific hypothesis. The purpose of this review is to provide an overview of the available innovative methods to study cancer metabolism, discussing experimental limitations, future directions and clinical applications.

## Metabolomics: a powerful tool in cancer research and treatment

Genomic and transcriptomic studies often fail to explain the complexity of a biological system, thus, leaving us with a huge gap in the map from genotype to phenotype. The approach that is closest to fill this gap is metabolomics, first introduced by Steven Oliver in a review article on yeast functional genomics published in 1998, and now widely recognized as a cornerstone to all of the systems biology^4,59,60^ (Fig. 1). Metabolomics is a high-throughput study of the metabolome which includes all the small molecules (50–1500 Da) with diverse physiochemical characteristics and dynamic range of abundance, commonly known as metabolites, within cells, biofluids, tissues or organisms.^61^ The identification of metabolites and their concentrations, unlike other omics measurements, directly represents the molecular phenotype. Metabolomics, more than any other methods, significantly revolutionized the field of cancer research; it is one of the most powerful omics techniques, mainly employed in cancer research to effectively detect metabolites whose level is affected by the neoplastic progression in a biological sample with a wide range of applications such as biomarker identification, drug discovery or development, clinical toxicology, nutritional studies, and quantitative phenotyping.^62,63^ As with any omics techniques, metabolomics is highly dependent on the availability and quality of public databases. In this regard, the biggest achievement in the field was reached in 2007 with the completion of the first draft of the human metabolome, the chemical equivalent of the human genome (Fig. 1). The Human metabolome database (HMDB) is a freely accessible electronic database (current version HMDB 5.0, https://hmdb.ca/) designed with the possibility to link data to other databases (KEGG, PubChem, MetaCyc, UniProt, and GenBank) thus combining chemical data, clinical information and molecular biology/biochemistry models.^64–68^ The database contains more than 220,000 metabolite entries including both water-soluble and lipid-soluble metabolites.

**Fig. 2:**
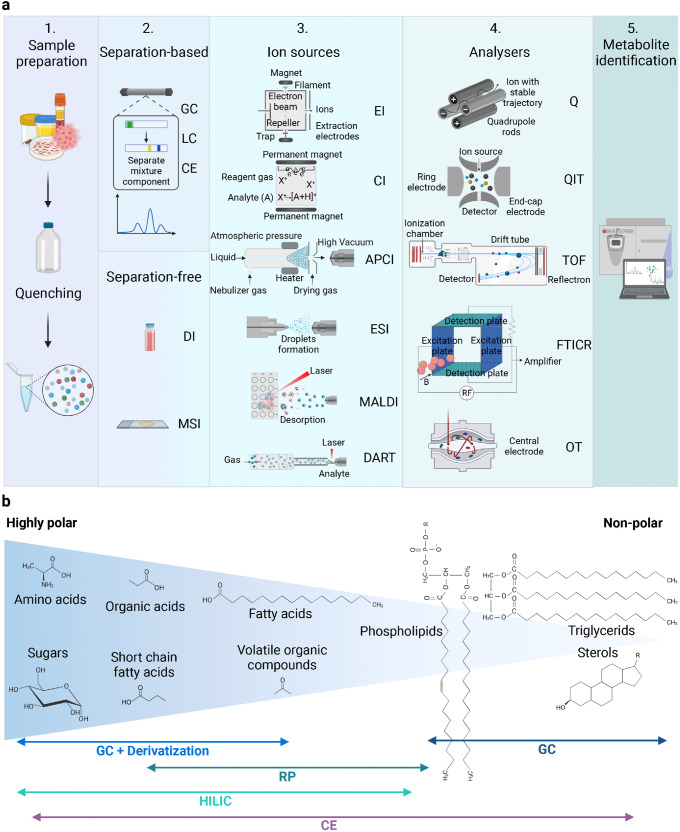
**a** Mass spectrometry (MS)-metabolomic workflow. 1. Samples preparation consists of metabolism quenching and metabolites extraction. 2. Metabolites may need a separation step with Gas chromatography (GC), Liquid chromatography (LC), Capillary electrophoresis (CE) or can be directly ionized in the Direct infusion (DI) and in the Mass spectrometry imaging (MSI). 3. Different ionization techniques can be employed: Electron impact ionization (EI), Chemical ionization (CI), Atmospheric pressure chemical ionization (APCI), Electrospray ionization (ESI), Matrix-assisted laser desorption ionization (MALDI) and Direct real-time analysis (DART). 4. Single (MS) or tandem (MS/MS) mass analysers can be alternatively employed to separate ions according to their *m/z*: Quadrupole (Q), Quadrupole ion trap (QIT), Time-of-flight analyser (TOF), Fourier transform ion cyclotron resonance (FTICR), Orbitrap (OT). 5. Data processing includes conversion of *m/z* values, detection, filtering, normalization and identification. **b** Schematic depicting the most suitable techniques to separate metabolites with distinct polarity. This figure was created with Biorender.com

However, despite increasing technological progress, none of the current analytical platforms can completely measure the whole metabolome. Reaching an adequate metabolome coverage remains a major challenge, which can be achieved only through a combination of approaches.^47,69–73^ The most critical step in a metabolomics workflow is the fast quenching of all metabolic pathways and the isolation of metabolites in order to completely block all enzymes and chemical activities and generate a stable extract with metabolite ratios and concentrations reflecting the levels of endogenous metabolites from the original living cell at a chosen time.^47,74^ In addition, the collection and extraction of the samples have to maintain the original analyte concentration, increase the instrument productivity, and lower the matrix effect in the analysis. Sample collection and storage can depend on target metabolites and are the source of the greatest variation; therefore, harmonization of these procedures is mandatory for a correct analysis.^75^ High-resolution spectroscopic techniques include Mass Spectrometry (MS) and Nuclear Magnetic Resonance (NMR) spectroscopy with specific and complementary applications in the field that may require a prior separation step and different ion sources^75^ (Fig. 2a).

### Metabolomics approaches: untargeted and targeted strategies

Metabolome profiling is typically performed through two different strategies: targeted or untargeted analysis (Table 1). The goal of targeted (or validation-based) metabolomic approaches is the identification and quantification of a relatively low number (generally less than one hundred) of known analytes, while the untargeted (or discovery-based) metabolomics is used for a more comprehensive analysis and relative quantification of metabolites; in this case, the analysis must be further validated with an orthogonal approach.^76^ In targeted metabolomics, by using native and isotopically labeled internal standards (IS) it is possible to reduce the false positives, thus more accurately identifying and quantifying the metabolites. Furthermore, the use of IS improves the matrix-induced ionization effects, thus enhancing the sensitivity. However, a limitation of targeted approaches is the partial metabolome coverage, which increases the chance of a wrong interpretation of the metabolic process of interest.^77^ Oppositely, the analysis with an untargeted metabolomic approach does not need any pre-existing knowledge or hypothesis even though, sample preparation and analytical methods affect the subset of metabolites detected, thus they must be chosen wisely. In untargeted metabolomics studies, two different approaches for data acquisition can be used: Data-dependent acquisition (DDA) and Data independent acquisition (DIA) mode. In DDA the metabolites with the highest signal intensity in a full-scan MS1 spectrum are selected to generate fragmentation patterns. The DIA approach integrates full MS1 with MS/MS fragmentation for all precursor ions, thus producing an extremely complex spectrum of fragmentation where decoding the connection between precursor and end-products is very hard. To perform metabolite identification, it is necessary to search the experimental MS1 or MS/MS data into the metabolome database. To increase the metabolome coverage, several libraries must be simultaneously interrogated therefore requiring a massive bioinformatic effort to reduce redundant matching. However, this process is further challenged by the fact that the nomenclature of metabolites is not fully standardized and varies widely across databases.^76^

**Table 1:** Features of untargeted and targeted metabolomics approaches

Metabolomics	
Untargeted	Targeted
Discovery strategy (hypothesis generating)	Validation strategy (hypothesis driven)
Global and comprehensive analysis	Subset analysis
High number of metabolites studied	Small number of metabolites
Correlated to database/libraries	Correlated to reference standards
Qualitative identification	Identification of metabolites known a priori
Relative quantification	Absolute quantification
Challenging data interpretation	Easy data analysis and interpretation
Samples preparation requires a global metabolites extraction	Samples preparation is metabolite-specific
Biomarker discovery	Biomarker validation
Global/untargeted screening	Pathway mapping targeted metabolomic profiling

### Separation-based MS techniques for metabolites investigation

To analyse the myriad of metabolites, which constitutes a biological sample, a separation through chromatographic systems may be necessary.^78^ The separation is required to avoid ion suppression or over-loading of the MS system, which causes a general loss of sensitivity. The interaction between the metabolites and the chromatographic system depends on the physical-chemical proprieties of the metabolites, the stationary phase and it determines the order of elution in the chromatographic system. Usually, lipids, volatile organic metabolites and derivative compounds are analysed by Gas chromatography (GC)-MS, originally described in 1959, while the analysis of the polar molecules is performed through Liquid chromatography (LC)-MS, introduced for the first time in 1974 (Figs. 1, 2a, b).^79^

#### Gas chromatography (GC)

GC coupled with MS is an analytical technique applied in metabolomics optimal for the analysis of small molecular substances (<650 Daltons). GC is a separative tool to quantitively and qualitatively measure volatile and thermally stable compounds. As a mobile carrier it uses an inert gas, such as nitrogen, helium, or hydrogen, while the stationary phase can be a liquid with low vapour pressure that coats the inner walls of a capillary (Gas liquid chromatography, GLC) or a solid that exhibits interactions with analytes by physical adsorption (Gas solid chromatography, GSC). Usually, the first choice is a stationary phase with similar polarity to the analytes while increasing the column temperature over time facilitates the elution of metabolites. Since most primary metabolites with a pivotal role in cancer metabolism such as lactic acid, pyruvic acid, malic acid, glucose, and palmitic acid have high boiling points, a derivatization step is necessary with the aim of making them volatile enough to be analysed by GC-MS. Among the derivatization protocols, the more employed ones use trimethylsilylation or thereof-like variants, which detach acidic protons from hydroxyl-, carboxyl-, amino- or thiol-group.^80–82^ The derivatization process is fast, with high yield, carried out under mild conditions, and break the molecular proton bridge bond making the boiling points lower and the metabolites more stable for GC-MS.^83^ Due to the complexity of biological samples, enzyme activity, and often the need to increase the concentration of low abundant molecules, the choice of the right procedure is dictated by the molecular target.^84,85^ With two-dimensional gas chromatography (GCxGC), a second independent separation is performed by importing the analytes eluted from the first column into the second column increasing both the peak capacity and the sensitivity thus facilitating the characterization of metabolites.^86,87^

#### Liquid chromatography (LC)

LC is a potent separative technique ideal for studying complex biological matrices, and it has been further optimized to ensure efficient, easy, and robust analysis. LC coupled to a mass spectrometer (LC-MS) revolutionized the field, allowing the characterization of non-volatile or thermally labile compounds with high molecular weight that were not possible to analyse with GC-MS. By reducing the diameter of the packing particles (3–50 µm) in the column and by employing high pressure to improve the mobile phase speed, it has been possible to make LC operating in a highly efficient mode, the so-called High-performance liquid chromatography (HPLC).^88^ To gain in selectivity and sensitivity some metabolomic studies have employed the Ultra-performance liquid chromatography (UPLC) system using columns with even smaller particle size (<2 µm) and higher pressure. Nevertheless, the low injection volumes and short column life do not make this technique widely used.^75^ A further development is the Ultra-high-pressure liquid chromatography (UHPLC), which is a further optimization capable of producing the best resolution and peak capacity for liquid chromatography known and therefore it is mainly applied to analyse complex samples, including biofluids.^88^ The analysis of metabolites with different physicochemical characteristics cannot be performed with a single stationary phase. Given its high reliability and robustness, Reversed-phase chromatography (RP) is one of the most widely employed technique, thanks also to the possibility of repeatable separations covering a broad-spectrum of chemical compounds. In a RP chromatographic system, the stationary phase consists of an organic species chemically bound to silica particles that form the chromatographic support, and it has a lower polarity compared to the mobile one. Non-polar columns, as C18, are ideal for metabolomic analysis because of their ability to separate semipolar compounds such as phenolic acids, flavonoids, glycosylated steroids, alkaloids, and other glycosylated species. However, other types of stationary phases are reported in RP chromatography like short alkyl chains (C8) and amides. In this type of separation, the mobile phase is polar (water, methanol, acetonitrile), and the elution can be isocratic (constant composition of the mobile phase) or gradient (variable composition); the latter mode is used for heterogenous samples because it improves separation and reduces the elution times of substances.^75^ Many biofluids analysed in metabolomic studies are aqueous thus containing many polar molecules that are almost not kept by the RP stationary phase. The separation of these polar analytes is mostly carried out by the Hydrophilic interaction liquid chromatography (HILIC) technique, which is a variation of the Normal phase (NP) chromatography, that uses a highly polar stationary phase (Fig. 2b).^75^ Many metabolites linked to tumorigenesis have hydrophilic chemical proprieties; in these cases, both ion-paring reagent-based RP chromatography and HILIC can be employed.

Reversed-phase ion-pair (RPIP) chromatography has a hydrophobic stationary phase and a mobile phase containing an ion-pair reagent, which consists of both hydrophilic and hydrophobic residues in a single molecular structure, added at low concentration. HILIC-based separation technique uses a hydrophilic stationary phase with which the water-soluble metabolites interact and a hydrophobic organic mobile phase, which has a percentage of solvent increasing over time enabling the elution of the metabolites from the column according to their affinity. The HILIC columns allow the retention of polar molecules and the fast elution of lipophilic compounds, which are usually difficult to elute from other types of columns and can therefore accumulate causing ion suppression due to background bleeding.^72,75^ Supercritical fluid chromatography (SFC) uses a supercritical fluid as mobile phase with temperature and pressure higher than a critical point. Among the most widely used eluents in the supercritical state is Carbon dioxide (CO_2_) is non-toxic, easily handled and environmentally friendly since it needs just little amount of organic solvent as auxiliary solvent.^89^

#### Capillary electrophoresis (CE)

CE is an analytical separative method applicable to a broad spectrum of chemical substances, primarily polar and charged ones (Fig. 2b). The most widely used separation modes for metabolomic analysis are Capillary zonal electrophoresis (CZE) and Capillary micellar electrokinetic chromatography (MEKC).^90,91^ CZE separates compounds according to the different electrophoretic mobility of the analytes, which is determined by their charge and size; the separation occurs in a capillary that contains a separation buffer, and it is subjected to an electric field.^92^ MEKC extends the functionality of CE to uncharged compounds; thus, the separation of the analytes happens through differential splitting between micelles and a surrounding aqueous solution. CE allows for different selectivity and higher efficiency than HPLC as well as shorter analysis times. However, this technique has low concentration sensitivity compared to HPLC, and, for this reason, sample pre-concentration is often necessary. CE-MS has great potentiality for microscale analytical technique, meeting the need of analytical methods to profile metabolism of (sub)microliters of samples.^93^ However, the CE-MS-based metabolomics is not widely diffused in comparison to other analytical separation approaches described above, as it is perceived as a technically demanding approach that suffers of low reproducibility and sensitivity.^92^

### Separation-free MS techniques for metabolites investigation

For fast and high-throughput analysis the option of a direct MS analysis, without any prior separation, may be particularly useful (Fig. 2a). In Direct infusion mass spectrometry (DI-MS) samples are directly injected or infused into high-resolution and high-accuracy MS, without a separation step. DI-MS enables rapid sample analysis, improving repeatability and accuracy among samples.^94–96^ However, isomeric compounds cannot be separated and contamination of the ion source by compounds residues is tedious but overcome by using chip-based nano-electrospray ionization (ESI, see “MS-Ion sources” paragraph below).^89^ Mass spectrometry imaging (MSI) is an interesting emerging approach to analyse metabolites in situ directly in organs, organoids or cells. MSI utilizes several ionization sources: Matrix-assisted laser desorption ionization (MALDI), Secondary ion mass spectrometry (SIMS), Desorption electrospray ionization (DESI), and Laser ablation electrospray ionization (LAESI). In MALDI-MSI, the laser targets the matrix-coated tissue surface to achieve tissue scanning.^97^ In SIMS, first a high-energy primary ion beam (Ar,^+^ Ga,^+^ and In^+^) hits the sample surface and then the secondary ions are assembled and analysed. DESI derives from electrospray and desorption ionization, and it exploits the conduction of electrosprayed charged droplets and solvent ions into the surface to analyse. LAESI is a mix between ambient ionization source grounded on mid-infrared laser ablation with charged droplets produced by Electrospray ionization (ESI).^89^

### The mass spectrometer: the metabolomics core instrument

The first mass spectrum of a molecule was measured in the 1910s by Joseph J. Thomson, who constructed the first mass spectrometer^98,99^ (Fig. 1). Initially, mass spectrometers were mainly employed by physicists to study the atomic weights of elements and isotopes and their respective relative abundance.^100^ This innovative technology was applied in biological settings in the 1940s, when heavy stable isotopes were used as tracers to study CO_2_ production in animals.^101^ Since that time, technological advances have increased the range of sample types that can be analyzed by MS. In recent years, MS has been widely used to investigate cancer-specific changes in the biomass composition of human tumours, including metabolic alterations since it qualitatively and quantitatively measures the compounds. A mass spectrometer is always composed by three elements: an ion source, a mass analyser, and a detector (Figs. 1, 2a).

#### MS-Ion sources

The analytes are first introduced into the ionization source, where they acquire either a positive or negative charge. At this point, the ions pass through the mass analyser and, on the basis of their mass-to-charge (*m/z*) ratio, arrive to distinct parts of the detector, where signals are generated and recorded by a computer system, which in turn graphically displays these signals as a mass spectrum. A hard ionization method like the Electron impact ionization (EI) renders GC-MS the right approach to identify metabolites. In EI a molecule in gaseous phase is ionized by collision with an electron flux of typically 70 eV of energy, giving rise to an excited molecular ion that can dissociate to generate fragment ions related to a structure. Structural information is extrapolated by disrupting the compounds and producing molecule-specific fragments. Then, the pattern of unique fragments is employed to identify metabolites by taking advantage of the available libraries.^102^ Chemical ionization (CI) is also commonly used in GC-MS analysis. It produces ions of the analyte of interest by ion/molecule reactions from ions of a reactant gas that is present in excess. This type of ionization is not the first choice in metabolomics since the obtained fragments are limited as well as the libraries for the following analysis. On the other hand, CI uses a soft ionization by applying low energy to the molecules and it can be used for unknown metabolite identification.^103^ Atmospheric pressure chemical ionization (APCI) is another soft ionization source which gives rise to some degree of fragmentation enabling structural characterization. Ions are formed in the gas phase using a corona discharge to ionize metabolites present in the aerosol, then the ions released in the gas phase are analysed with the analyser. APCI is commonly used to analyse small polar and non-polar compounds that are poorly ionized by ESI (see below) and it is commonly coupled with HPLC. APCI is a useful tool for lipidomic; in particular, it generates ions from large neutral molecules such as triacylglycerols that are still a challenge to analyse with other techniques.^104–106^ Electrospray ionization (ESI) is the more widely used ion source in metabolomic studies; it generates ions from metabolites out of a solution and it is coupled to liquid-based separation techniques like LC, CE or DI. Its soft ionization produces vast number of ions through charge exchange in solution and forms intact molecular ions that aid the initial identification. This ionization technique provides a sensitive, robust, and reliable tool to study at femto-mole quantities non-volatile and thermally labile analytes that are difficult to analyse with other conventional techniques. Clusters of charged droplets consisting of analytes surrounded by many solvent molecules are produced by introducing a fine spray of a liquid solution of molecules into an electric field (2–4 kV) outstanding between the capillary and the counter electrode of the MS inlet at atmospheric pressure. Then, a flow of nitrogen (drying gas) enhances the evaporation and elimination of solvent from the charged analyte. The droplets size decreases due to the evaporation of solvent and the charge density increases till the coulombic explosion of the droplet. At this point, the resulting analyte ions enter the MS *via* electrostatic lenses.^75,107,108^ Matrix-assisted laser desorption ionization (MALDI) is a tool for soft ionization and transfer of complex samples, which are co-crystallized with an organic matrix on a metal target, from the solid phase to the gas phase. A pulsed laser excites the matrix and induces a fast heating of the molecules and the desorption of ions to the gas phase; the selection of the matrix is a fundamental aspect that affects the experimental results. The most used MALDI matrices are α-cyano-4-hydroxycinnamic acid (CHCA), 2,5-dihydroxybenzoic acid (DHB) and 3,5-dimethoxyl-4-hydrocinnamic acid (SA).^97^ This technique is suitable for high molecular weight analytes, it is fast, consumes little amount of sample and has high tolerance towards impurities (e.g., salts).^109^ By contrast, it must have low vapour pressure and the pulsed nature of the laser limits compatibility with the analyser. In fact, normally, the MALDI is associated to a MS capable of measuring a full mass spectrum without scanning a mass range.^89^ MALDI-MS is a potent analytical tool and a valuable method in cancer research and tissue imaging, due to its ease of use and high mass resolution.^110–114^ Direct real-time analysis (DART) is another recent ionization technology that allows metabolites to be analysed without sample preparation and at atmospheric pressure. In DART, there is first the thermo-desorption of condensed-phase analytes by a stream of a hot gas (such as helium, argon or nitrogen), which carries active species derived from a plasma discharge. Then, the ionization enables the acquisition of respective mass spectra. DART is used with small molecular compounds with minor cross-contamination and provides a simple and high-throughput analysis.^89,115^

#### MS analysers

Improving resolution and sensitivity is the main goal of metabolomics studies. Nevertheless, often the two parameters are mutually exclusive, and this remains the main limitation of metabolomics. Single (MS) or tandem (MS/MS) mass analysers can be alternatively employed with distinct output and final resolution. The single-configuration mass analysers mostly used in metabolomics are the Quadrupole (Q), Quadrupole ion trap (QIT), Time-of-flight (TOF), Fourier transform ion cyclotron resonance (FTICR), and Orbitrap (OT) (Fig. 1a).^116^ Quadrupole (Q) has four parallel cylindrical rods, which are responsible for filtering the sample ions based on their *m/z*.^117^ Ions are separated according to their trajectory stability in the oscillating electric field applied to the rods. By continuously modulating the applied voltages, it is possible to select a particular *m/z* or scan for a range of *m/z* values. Quadrupole ion trap (QIT) is a variant of the quadrupole analyser with three electrodes (an annular electrode placed between two hemispherical inlet and outlet electrodes) to trap and accumulate ions in a small cavity, called ion trap. The mass spectrum is obtained by varying the electric potential so that the ions are sequentially ejected from the trap toward the detector according to an increasing *m/z* value.^118^ Time-of-flight analyser (TOF) consists of a field-free drift chamber held under high vacuum through which ions run. The ions are separated according to their velocity, and they reach the detector at separate times since they possess the same kinetic energy but a different velocity depending on the *m/z* ratio. The same analytes can be desorbed at slightly different times, therefore, a reflectron composed of sequential electrodes is placed at the end of the TOF resulting in an increased mass resolution and accuracy of TOF analysers.^119^ Fourier transform ion cyclotron resonance (FTICR) is a high resolution and high accuracy but also very expensive analyser, which works by applying a strong magnetic field (9–15 T), thus the ions assume a circular motion in a plane perpendicular to that of the magnetic field (cyclotron). Ions start to rotate with an angular frequency inversely proportional to *m/z*. By applying a radiofrequency voltage pulse corresponding to the angular frequency of a specific analyte, this ion with a specific m/z value absorbs energy and produces a cycloidal path moving with a wider orbit. The way the excited ions move creates the image current measured on electrodes. Since the ions oscillate as a function of their *m/z*, by measuring the image current during time and using a Fourier transform (FT), it is possible to extrapolate the *m/z* values. By applying a scan of voltages, a complete spectrum is obtained.^120,121^ Orbitrap (OT) traps in an electrostatic field positive ions because of their attraction to the inner electrode (set at about –3200 V). The ions start to rotate around the inner electrode and together oscillate along the *z*-axis according to their *m/z* ratios. An image current is detected and converted by a FT to obtain a mass spectrum with the frequency and the intensity of each ion.^122,123^ Quadrupole and ion trap analysers are extremely sensitive but limited in resolution, whereas TOF, FTICR and OT offer the highest mass resolution. Mass analysers in a tandem configuration include diverse types of analysers.^124^ Triple quadrupole (TQ) and Triple-quadrupole ion trap (QTrap) analysers, which have high sensitivity and selectivity, are the most common MS-spectrometers coupled to LC and employed in targeted metabolic studies. In contrast, the high mass-resolving capacity of Quadrupole-TOF (Q-TOF), Linear-quadrupole ion trap-Orbitrap (LTQ-Orbitrap), and FTICR analysers is ideal for globally profile and to identify metabolites. GC is mainly coupled with single quadrupoles or TOFs, but novel instruments also exploit QTOF or TQ MSs.^116,125,126^

### Nuclear magnetic resonance (NMR) spectroscopy: an alternative approach in metabolomics

NMR spectroscopy, originally described by Isidor Rabi in 1938 and later used for the analysis of liquids and solids by Felix Bloch and Edward M. Purcell in 1946, is a consolidated approach for the analysis of cancer metabolism^69,127^ (Fig. 1). This analytical tool measures the chemical shifts of atomic nuclei with non-zero spin (i.e. ^1^H, ^31^P and ^13^C), which are dependent on the atom environment in a chosen analyte, and enable detecting and elucidating the structure thanks to shifts in the magnetic resonances.^128^ NMR spectroscopy allows the characterization of new compounds and it does not require sample destruction, chromatographic separation, sample treatment, or chemical derivatization.

NMR is highly automatable and, if the same instruments and analytical strategies are employed, the reproducibility between different laboratories is guaranteed.^129^ Unlike other metabolomic platforms, NMR is not limited to the analysis of biofluids or tissue extracts, but it can be employed for the study of any biological sample, including a solid or semi-solid tissue or organ^130–133^ *via* either the solid-state NMR (ssNMR) or the magic-angle sample spinning (MAS-NMR).^134^ NMR allows live analysis coupling the imaging and the metabolic profiling with the Magnetic resonance spectroscopy (MRS) and the Magnetic resonance imaging (MRI). Moreover, it is widely employed to profile metabolic fluxes in real time.^129^ The primary limitation of this technique is the low sensitivity which is 10 to 100 times lower in comparison to LC-MS and GC-MS. A significant signal enhancement has been achieved by increasing the magnetic field, using a cryo-probe and the processing of digital signals; however, most of the low-concentrated small molecules are still undetectable with NMR. In order to make NMR analysis high-throughput, innovative fast and ultrafast multidimensional NMR approaches have been introduced while NMR sensitivity has been enhanced by altering the nuclear polarization with the Dynamic nuclear polarization (DNP).

However, the NMR applications in metabolomics are limited mainly because NMR spectrometers are more expensive than many other analytical instruments including mass spectrometers and they need experienced users and vast laboratory infrastructure with ad hoc non-vibrational floors and specific area isolated from magnetic and radio frequency interference.^135^

### Metabolic flux analysis (MFA)

**Fig. 3:**
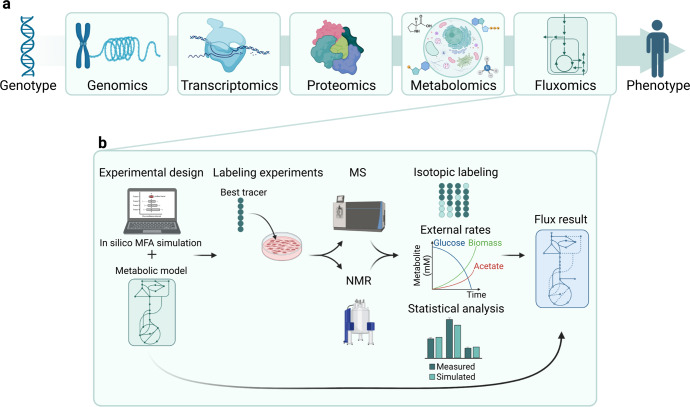
**a** Fluxomics is the omics approach that gets closer to the phenotype. **b** Experimental workflow of a standard Metabolic flux analysis (MFA) experiment. The first step is the experimental design and the definition of the best tracer and metabolic model by in silico analysis. Cells or tissues are incubated with the tracer and samples are analysed by either MS or NMR. The isotope tracing is analysed considering the metabolites external rates and the statistical value. The results are thus integrated into the original metabolic model. This figure was created with Biorender.com

Global metabolomics analyses are robust techniques that allow the identification of tumour-associated metabolic liabilities; however, often they fail to reveal the plasticity and dynamism of metabolic pathways, providing only a static snapshot of cancer cell metabolism. Metabolic flux analysis (MFA), using stable-isotope-labelled substrates, can determine flux rates by tracing the isotope enrichment of particular atoms on downstream metabolites, thus really connecting omics analysis and phenotypes (Fig. 3a).^72,136^ As depicted in the workflow of Fig. 3b, the first step is the design of the experimental setting that includes the in silico identification of the optimal tracer for the highest flux resolution and the substrate labelling. Following administration of the stable-isotope tracer, samples are analysed both for isotopic labelling and external rates that takes into account substrate uptake and product secretion. The metabolite labelling can be measured either by MS or NMR and integrated into the metabolic network model to which the labelling measurements are fit and must include all relevant reactions and their respective carbon atom transitions. The network is constructed based on information from metabolome databases.

Two different experimental settings are mainly used in MFA: stationary or non-stationary. The stationary design deals with metabolites at their basal state: the labelling is not linked to the analyte’s concentration thus simplifying the flux measurement and allowing the study of the involvement of different metabolic routes to the final amount of a given metabolite. By contrast, to analyse the flux to a downstream pathway a non-stationary design should be employed. In this setting, a labelled substrate is applied for a brief time, in order to detect the label incorporation into downstream molecules.^137^ According to the pathway of interest, it is possible to choose among substrates that can be uniformly or positionally labelled. Generally, the first type of substrates is employed to analyse complex fluxes, while positionally labelled substrates allows the detection of small changes into key node of metabolic pathways. 1,2–^13^C_2_ glucose is used to accurately quantify glycolysis, PPP and gets a general picture of the energy metabolism of a tumour cell, while U-^13^C_5_ glutamine is the best substrate for the measurement of the TCA cycle.^128^ In cancer metabolism research, the most common labelling is with ^13^C for substrates as glucose, glutamine, pyruvic acid, lactic acid, palmitic acid, amino acids, and acetic acid.

Carbon flux distribution into downstream and interconnected metabolic pathways of ^13^C labelled substrates are determined by MS or NMR techniques; it is thus possible to understand how dependent the biosynthesis of a metabolite is to a specific source of carbon and what is the flux into downstream metabolic routes. To measure specific metabolic activities there are many tracers. 1–^2^H and 3–^2^H-glucose can be employed to quantify the biosynthesis of NADPH from the Oxidative pentose phosphate pathway (OxPPP)^138,139^ and Nicotinamide adenine dinucleotide (NAD) synthesis-breakdown fluxes.^140^ MFA is an extremely useful method to identify metabolic shifts, but both the measures and the bioinformatics and statistical analysis are extremely complex requiring skilled workforce and cutting-edge infrastructures.^128^

### Translational metabolomics: from bench to bedside

The substantial technological, methodological and computational advancements in metabolomics platforms and the growing accessibility of the technologies over the last 40 years have led to the development of personalized metabolic profiling that in concert with personalized genomics, represent the foundation of personalized medicine. Metabolomics can be employed to analyse a wide range of samples (i.e. tissue, biofluids, cells), thus spacing outside the academic institutions and finding applications in many different disciplines including human health, wellness and food chemistry.^141^ The new metabolomic platforms have provided opportunities for cancer screening, diagnosis and treatment.^63,142–145^ The most successful example of application is the employment of metabolomics to discover and validate biomarkers for tumour progression and metastasis in different sample types including plasma or serum,^146–156^ urine,^157–160^ saliva^161–164^ and cerebrospinal fluid.^165–167^ Metabolomics can be used for non-invasive diagnosis and prognostic evaluation of cancer^147,168–171^ and for tumour subtyping.^168,172–175^ Moreover, metabolomics constitutes a cost-effective and productive platform for monitoring anti-cancer treatment effects, both as a predictive measure of efficacy and as a pharmacodynamic marker as well as for evaluation of drug resistance.^176–181^ One of the most interesting and recent application is the use of metabolomics as an alternative method to the most traditional immune and biochemistry assays to investigate the metabolic alterations in response to immunotherapy.^182^ Employing metabolic profiling by metabolomics, to evaluate the immune responses in cancer patients, is an innovative area of cancer research with the potential for identifying host immune factors that would prevent the effective anti-tumour immunity thus compromising the clinical output.^183–192^

## Extracellular flux analysis (EFA)

**Fig. 4:**
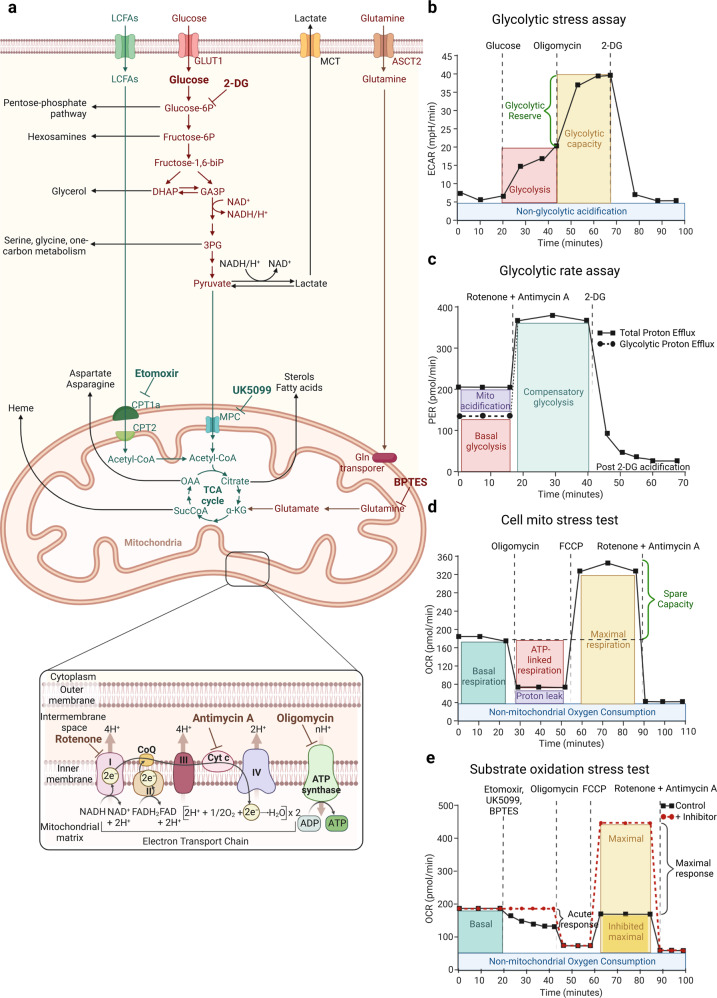
**a** Key metabolic pathways governing cancer cell growth that can be measured by Extracellular flux analysis (EFA). **b** Glycolysis stress assay is performed by serial injections of Glucose, Oligomycin and 2-deoxyglucose (2-DG) to get as a read out glycolysis, glycolytic capacity, the glycolytic reserve and non-glycolytic acidification measurements. ECAR stands for extracellular acidification rate. **c** The Glycolytic rate assay reports multiple key parameters, such as basal glycolysis, compensatory glycolysis achieved by shutting down mitochondrial respiration with rotenone and antimycin A. Proton efflux rate (PER) is a quantitative measure of protons extruded into the extracellular medium during glycolysis. **d** In the Cell mito stress test, mitochondrial respiration is measured by quantifying the Oxygen consumption rate (OCR). Cells are sequentially exposed to oligomycin, Carbonyl cyanide 4-(trifluoromethoxy) phenylhydrazone (FCCP) and rotenone and antimycin A thus allowing the measurement of the basal and maximal respiration and spare respiratory capacity. **e** The Substrate oxidation stress test measures the contribution of Long-chain fatty acids (LCFAs), glucose/pyruvate and glutamine as primary substrates that fuel mitochondrial metabolism by using specific inhibitors in combination with a standard Cell mito stress assay. Etomoxir inhibits the Carnitine palmitoyl transferase 1a (CPT1a), UK5099 blocks glucose and/or pyruvate through inhibition of the Mitochondrial pyruvate carrier (MPC) and BPTES inhibits glutamine through Glutaminase-1 (GLS-1). This figure was created with Biorender.com

Extracellular flux analysis (EFA), with the Agilent Seahorse XF analyser as leading technology counting more than 5000 peer-reviewed publications, is the most common and feasible method to broadly quantify the bioenergetic activity of live cells, organoids or tissues^193^ (Fig. 4a). Sketchily, EFA quantifies Oxygen consumption rate (OCR) as a quantitative measurement of mitochondrial electron transport rate named mitochondrial OXPHOS and the Extracellular acidification rate (ECAR) as a read out of glycolysis. The instrument performs live measures of OCR and ECAR by taking just few μL of medium from monolayer of cells in microplates. Two sensors installed into fibre-optic probes quantify oxygen level and medium pH for several minutes, then the software extrapolates the OCR (pmol/minute) and ECAR (mpH/min) quantifications. The versatility of the instrument is given by a continuous and systematic temperature regulation that controls the extracellular microenvironment and an integrated drug delivery equipment for serial injection of up to four metabolic inhibitors to each well at pre-set time points, thus allowing measurement of metabolic capacity and adaptation.

Glycolysis is a hallmark of cancer cells and its analysis together with the associated metabolic rewiring are considered reliable diagnostic means for detection and prognosis of malignant phenotypes and to lead to the identification of new biomarkers and therapeutic strategies.^194,195^ The Glycolytic stress assay is the easiest method to gain a qualitative overview of the bioenergetic phenotype (Fig. 4a, b). In this assay, first ECAR is measured right after incubation of the cells in a medium in which glucose was depleted. This is followed by a first injection of glucose until saturation causing a rapid extrusion of protons into the medium quantified by a rise in the ECAR. This glucose-induced response is considered as basal glycolysis rate under normal conditions. Injection of oligomycin, a Complex V (ATPase) inhibitor, interferes with mitochondrial respiration by blocking the proton channel of the ATP synthase thus shifting the cellular bioenergetics towards glycolysis, which is detected by an increased in ECAR: the so called, cellular maximum glycolytic capacity. The last injection is with 2-deoxy-glucose (2-DG), a synthetic glucose analog that blocks glycolysis through competitive binding to glucose Hexokinase (HK), which causes a drastic decrease in ECAR and serves as positive control of ECAR shifts as read out of glycolysis. The measurement of ECAR, before the injection of glucose, is the non-glycolytic acidification (due to any intracellular metabolic activity other than glycolysis) while the difference between the glycolytic capacity and the glycolysis rate allows the calculation of the glycolytic reserve. At pH of 7 the physiological conversion of glucose into lactate causes protons release and acidifies the medium, thus providing a quantitative assessment of the glycolytic rate. However, this quantification does not consider that the final ECAR is the result of two processes: glycolytic and respiratory acidification. More in detail, CO_2_ derived from the TCA can be spontaneously or enzymatically hydrated to carbonic acid, H_2_CO_3_, that dissociates to HCO_3_^−^ and H^+^ in a medium with physiological pH. The conversion of one molecule of glucose into lactate yields 2 lactate^−^ and 2 H,^+^ whereas the complete oxidation of one glucose to CO_2_ generates molecules of HCO_3_^−^ and 6 H^+^ molecules; as a consequence, the extracellular acidification when a glucose molecule is oxidized to CO_2_ is three times greater than when it is converted to lactic acid. The ratio of glycolytic and respiratory acidification is significantly affected by the experimental conditions, such as cell type and media composition, and it can fluctuate from nearly 100% glycolytic acidification to nearly 100% respiratory acidification. Given the linear correlation between O_2_ consumption and CO_2_ production during OXPHOS, the Glycolytic rate assay implements the previous analysis by calculating the contribution of mitochondria/CO_2_ to extracellular acidification and subtract the CO_2_-dependent acidification from the total Proton efflux rate (PER). The resulting value, named “glycoPER”, is a quantitative and reliable measure of protons extruded in the culture medium during glycolysis (Fig. 4c).

Complementary, glycolytic flux can be measured by quantifying glucose uptake and lactic acid excretion: several commercially available methods based on colorimetric and fluorimetric read-outs are optimized to quantify glucose and lactate levels within the extracellular media. In addition, measurement of the activity of the three rate-limiting glycolytic enzymes [HK, Phosphofructokinase-1 (PFK-1), and Pyruvate kinase (PK)] is crucial for understanding the energetic metabolic status of the cells.^196^

The Cell mito stress test is a common EFA to fully characterize mitochondrial respiration based on the quantification of the OCR after interference with the complexes of the Electron transport chain (ETC) (Fig. 4a, d). After the initial basal OCR measurement, in the stress test, cells are exposed to oligomycin, which affects ATP synthase, causing a drastic decrease in mitochondrial respiration or OCR. Subsequently, electron flow through the ETC is simulated by the injection of the uncoupling agent Carbonyl cyanide 4-(trifluoromethoxy) phenylhydrazone (FCCP) so that oxygen consumption by complex IV reaches its maximum. This increase in OCR is used to extrapolate the cellular spare respiratory capacity, by definition the difference between maximal respiration and basal respiration, which indicates the capability of the cells to cope with stress and respond to changes in energetic demand thus indicating the cellular fitness. The third injection is a mixture of rotenone and antimycin A, respectively complex I and complex III inhibitors. The two inhibitors completely shut down mitochondrial respiration and allow the quantification of non-mitochondrial respiration caused by processes independent from mitochondria. This assay is particularly useful in pharmaceuticals pipelines; indeed, damages to mitochondrial function are important indicators of drug-mediated liver, cardiac and neurological disfunctions. Therefore, the integration of this test into the toxicology studies would allow a better evaluation of the drug side effects and improve the chemical substance synthesis upstream the later development steps.^197–201^

To generate ATP via OXPHOS, the nutrients taken up from the extracellular environment are ultimately oxidized *via* the TCA cycle and the ETC. The Substrate oxidation stress test is a useful method to evaluate the contribution of the three primary substrates in generating energy: Long-chain fatty acids (LCFAs), glucose/pyruvate and glutamine. The assays combine the Cell mito stress test for the interrogation of mitochondrial function (described above in this paragraph) and anaplerotic pathway specific inhibitors such as Etomoxir for LCFAs by inhibiting the Carnitine palmitoyl transferase 1a (CPT1a),^202–204^ UK5099 to block glucose and/or pyruvate by interfering with the Mitochondrial pyruvate carrier (MPC)^205–207^ and BPTES that blocks the Glutamine through Glutaminase-1 (GLS-1).^208,209^ When we are in the situation of substrate oxidation, the basal and especially the maximal respiration rates are significantly affected by the capacity of the cells to transport and utilize the available substrates revealing dependence on a specific metabolic pathway (Fig. 4a, e).

These EFA assays have significantly facilitated the identification of novel metabolic liabilities to genetic manipulation, pharmaceutical interventions and, thanks to the screening assays that allow the simultaneous analysis of up to 80 compounds in one plate, the systematic analysis of metabolic dependence of cancer-derived cells and organoids.^210^ However, cell media composition, cell density, inhibitor concentration and post-run normalization strategy (i.e., cell count or protein/DNA quantification) can dramatically impact the interpretations of the analysis as well as the interlaboratory standardization. OCR and ECAR analyses do not reflect individual pathway activity, but several variables can influence their proportionality to the metabolic processes they are meant to represent. Therefore, the interpretation of these analyses cannot negate the activity of secondary bioenergetic pathways; however, these assays can be usefully employed to compare samples and categorize the cells whether they are broadly more glycolytic or oxidative. Moreover, OCR and ECAR analyses can be complemented with additional techniques such as single-cell imaging using commercially available fluorescent dyes (see “Fluorescent metabolic probes” paragraph below) and ultrastructural morphological and morphometric analysis. For example, Transmission electron microscopy (TEM) evaluation of mitochondria (i.e., mitochondrial length, inner/outer membrane ratio, cristae extension, width, and junction diameter) is a valuable and complementary tool to investigate mitochondrial morphology and functionality.^211^

## Single-cell metabolic analysis

**Fig. 5:**
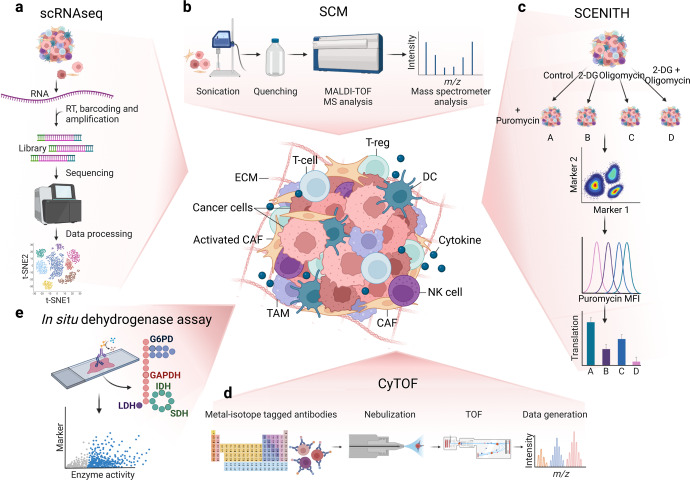
Global overview of the cutting-edge techniques available to investigate the Tumour microenvironment (TME). **a** Single-cell RNA-sequencing (scRNAseq) representative workflow: individual cells are isolated, lysed and barcoded before retro-transcription, library amplification and sequencing. **b** Single-cell metabolomics (SCM) includes single cells isolation, sample processing, quenching of metabolism and metabolomics. **c** In the single-cell energetic metabolism by profiling translation inhibition (SCENITH) the sample is divided into 4 and either left untreated or treated with 2-deoxyglucose (2-DG), oligomycin and both inhibitors. The addition of puromycin allows protein synthesis quantification coupled with flow-cytometry phenotyping. **d** In Cytometry by Time-of-flight (CyTOF), the cells are labelled using stable heavy metals, nebulized, and vaporized to form ion clouds and analysed by a Time-of-flight (TOF)-MS. **e** With the In situ dehydrogenase activity assay, the activity of G6PD, GAPDH, LDH, IDH and SDH is coupled with immune-staining to distinguish single cells directly on a tissue slide. This figure was created with Biorender.com

Up to date, most of the findings regarding cancer metabolism have been achieved using cell culture models and in vivo measurements obtained from bulk tumours. Indeed, one of the biggest limitations of standard metabolomics and EFA is that they do not allow the multiplexing of metabolic state and phenotyping. The result is always shaped by genetic and environmental factors for each cell and thus largely incompatible with heterogeneous cellular population. Moreover, standard cell culture media do not resemble the human physiological nutrient milieu and nutrient availability in a tumour microenvironment significantly modulates metabolic dependencies.^212,213^ The emergence of RNA-sequencing (scRNAseq) provided the unprecedented opportunity to interrogate genomic profiles of tumours with high resolution.^214–222^ Detection of changes in the expression of metabolic genes is a valuable tool to extend our comprehension of the metabolic rewiring of cancer and to identify metabolic vulnerabilities.^223,224^ For this analysis, individual cells collected in sub-microliter droplets are sorted into multiwell plates using microfluidic devices; after lysis, the cells are barcoded in order to assign sequencing reads to each cell (Fig. 5a). This technological advance was achieved thanks to the possibility of capturing and sequencing exceptionally small quantities of RNA. scRNAseq studies showed that malignant cells globally up-regulate genes involved in almost all functional categories of metabolic pathways suggesting a high metabolic plasticity that confers great adaptability to different genetic and environmental factors. This global transcription rewiring of metabolic genes suggests that cancer cells reserve more transcriptional resources for the expression of such those genes with consequently increased fluxes for most metabolic reactions.^225^ Single-cell, high-dimensional profiling allowed the identification of most effective immunotherapeutic approach, the discovery of biomarkers for early-stage of the disease and drug-resistance mechanisms in a variety of cancer settings. Indeed, scRNAseq analysis revealed a high-resolution snapshot of drug-resistant cells to Immune checkpoint inhibitors (ICIs), thus providing the rational for further investigating cell-cell communications and drug effect.^226–230^

The recent technological advances offered the opportunity to analyse the complexity of biological systems at the single-cell level also in metabolomics.^231^ Single-cell metabolomics (SCM) provides a snapshot of all metabolites, intermediates, and end-products of cellular metabolism within a biological system decoding the biochemical heterogeneity within cells.^232,233^ As previously discussed for bulk metabolomics, the biggest challenge in preparing single-cell samples is avoiding, or at least reducing, the impact on cellular metabolism due to the sample processing. The standard methods for single-cell sample preparation are the Fluorescence-activated cell sorting (FACS) and the microfluidic arrays that maintain intact cell morphology or extraction *via* an Atomic force microscopy (AFM) probe that keeps only the metabolites from the cell in the probe.^234,235^ Cellular metabolism should be quenched and can be analysed by MS-based technologies (Fig. 5b). Previous genomic analysis targeting metabolic genes at the single-cell level has revealed that individual malignant cells have elevated metabolic activity and variations that were not observed in bulk tumour studies. As for scRNAseq, SCM helps in dissecting cell heterogeneity^236^; SCM is particularly useful in oncology for disease and metastasis detection, precision drug design and drug assessment and toxicity.^237^ Moreover, SCM could be employed for profiling rare or Circulating tumour cells (CTCs)^238–240^ as well as for cancer subtypes discrimination and new therapies development.^225,241–244^

In addition, several cytometry-based methods that combine metabolic analysis with single-cell phenotypes have been developed. Fluorescent metabolic probes and analogues of metabolites can be analysed by flow cytometry or microscopy enabling single-cell resolution and they are often used for metabolic pre-screening assays since they are fast, relatively cheap, and easily adaptable to different experimental settings. Some examples are: 2-NBDG (2-(N-(7-Nitrobenz-2-oxa-1,3-diazol-4-yl)Amino)−2-Deoxyglucose), a fluorescent indicator to monitor glucose uptake into living cells; BODIPY (4,4-Difluoro-1,3,5,7,8-Pentamethyl-4-Bora-3a,4a-Diaza-s-Indacene) employed as a synthetic precursor to a wide variety of fluorescent phospholipids; CM-H2DCFDA, a chloromethyl derivative of 2,7-Dichlorodihydrofluorescein diacetate (H2DCFDA), useful indicator for ROS in cells; cystine–Fluorescein isothiocyanate (FITC) conjugate to monitor the uptake and accumulation of the amino acid; Monodansylcadaverine (MDC) to track autophagic vacuoles; JC-1 (5,5′,6,6′-tetrachloro-1,1′,3,3′-tetraethylbenzimi-dazolylcarbocyanine iodide), a carbocyanine dye that can be used as a ratiometric indicator of mitochondrial potential and MitoTracker dyes that are cell permeable probes containing a mildly thiol-reactive chloromethyl moiety for mitochondrial labelling.^245–249^

In 2020, an innovative flow-cytometry-based method to profile the energetic metabolism with single-cell resolution has been reported (Fig. 1). Single-cell energetic metabolism by profiling translation inhibition (SCENITH) measures metabolic profiles based on metabolism-dependent translation rates and puromycin incorporation into nascent proteins.^250^ As for EFA, incubation of given samples with on-target inhibitors enables to functionally estimate the glucose and mitochondrial dependence, glycolytic, fatty acid and amino acid oxidation capacity. It is possible to employ puromycinylation detection in combination with multiparametric flow-cytometry analysis thus analysing at single-cell complex and heterogenous samples^251–253^ So far, SCENITH has been applied to the analysis of ex vivo whole blood and human tumour biopsies. Interestingly, the combination of SCENITH and scRNAseq analysis of renal carcinomas and juxta-tumoral tissues succeeded in the correlation of the metabolic profile and the metabolic gene expression.^250^ Moreover, SCENITH can be employed in many other tumour settings and physio-pathological conditions including for example the comprehension of cell death pathways that have important and multiple connections with metabolism and redox imbalance^254–259^ (Fig. 5c).

Other powerful methods are based on single-cell proteomic analysis. In 1994 Tsutomu Nomizu and his collaborators discovered that it was possible to nebulize, dry and ignite individual cells in a hot plasma in order to create ion clouds that could be detected by emission spectrometry. This is the first real MS experiment of single cells.^260,261^ Over the years the technology had a big evolution until 2007, when Scott D. Tanner, inspired by the flow-cytometry technology, invented the mass cytometry, also termed Cytometry by Time-of-flight (CyTOF), which is the most promising technology for high-dimensional and high-throughput protein (and metabolic) single-cell analysis (Fig. 1). CyTOF uses non-biologically available metal isotopes, with concise mass spectrometry parameters, in replacement to standard fluorescent labels, normally employed in flow-cytometry. For the staining, the cells are incubated with a mixture of probes/antibodies tagged with unique non-radioactive heavy metal isotopes. Afterwards the single-cell suspension passes through an argon (Ar) plasma, by which the sample is atomized and ionized thus converting each cell into a cloud containing ions of the elements present inside or on that cell. Low-mass ions derived from each cloud are extruded by a high-pass optic thus generating a cloud containing only ions associated to the isotope-conjugated probes. Ions are then separated by *m/z* in the TOF chamber, with subsequent amplification and conversion into electrical signals. Up to 50 parameters can be studied simultaneously, overcoming all pitfalls associated with overlapping emission spectra that are normal for fluorescent-based analysis. Mass cytometry has tremendous potential for the analysis of highly heterogenous clinical samples as well as for the diagnosis and for studying the evolution of malignant disorders.^262,263^ The applications include the study of cell phenotype and function, signalling networks, apoptosis, cell cycle analysis, and many other complex biological processes.^264–269^ In addition, CyTOF analysis have been employed in several clinical research trials around the world to investigate multiple areas of human disease to understand and improve prevention and therapeutics. The biggest limitation that remains is inherent to the complexity of the data analysis which requires advanced biostatistical and bioinformatic skills and often makes its application in a clinical setting very complicated (Fig. 5d).^270,271^

Another single-cell proteomic method is Met-flow, a high-parameter flow-cytometry technique that uses specific antibodies against key metabolic proteins that are crucial in their representative pathway such as enzymes and transporters of the PPP, TCA, fatty-acid synthesis and oxidation, OXPHOS, and amino acid metabolism. Using 10 metabolic proteins, Met-flow can define cell subsets comparably to the resolution obtained by 500 genes by scRNAseq.^272^

Cellular metabolism in the Tumour microenvironment (TME) is affected by both genetic and environmental variables including intrinsic features of the tissue of origin, somatic mutations that appear during the tumour progression, the local nutrient environment, and the complex network of interactions between tumour, stromal and immune cells. Therefore, defining the metabolic signatures of the cells within their native microenvironment is mandatory to identify metabolic intercellular patterns. To this aim, new methods that employ metabolic imaging to quantify enzyme activity of single cells within tissue slices have been developed.^273^ With the in situ dehydrogenase activity assay, the quantification of the activity of five enzymes catalyzing key steps in the main metabolic routes [Glucose-6-phosphate dehydrogenase (G6PD) in the PPP, Glyceraldehyde 3-phosphate dehydrogenase (GAPDH) in glycolysis, Lactate dehydrogenase (LDH) in lactate fermentation, and IDH and SDH in the TCA cycle] is combined with staining to distinguish and characterize cell populations. Enzyme activities are quantified on different tumour tissue cryosections and the amount of formed product is measured at optimal and constant assay conditions, including saturating substrate and co-factor levels. The resulted values depict the amount of active enzyme in the analysed samples, associated with a phenotype and to a localization. The interpretation of the results must consider that the activity of these distinct dehydrogenases is quantified at saturated substrate concentrations; therefore, they do not reflect a precise in vivo setting but can then be used to compare different samples (Fig. 5e).

## Functional genetic screening

**Fig. 6:**
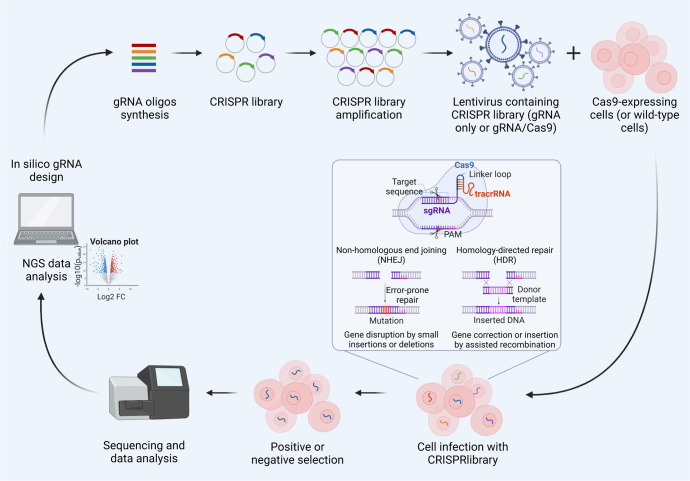
General workflow for screening using CRISPR/Cas9 libraries. The guide RNA (gRNA) library is synthetized after in silico optimization and cloned into plasmids for the amplification. A library of lentiviruses is then produced and used to infect the cells harbouring or not the Cas9 enzyme. A positive or a negative selection can be applied to identify specific phenotypes and next-generation sequencing is used to determine which genes are disrupted and which are not. This figure was created with Biorender.com

Combining functional screening with metabolic profiling gives the incomparable opportunity to identify context-dependent vulnerabilities that may be therapeutically targeted. Recent innovations in genome editing technology and the advent of the clustered regularly interspaced short palindromic repeat (CRISPR)/CRISPR-associated protein 9 (Cas9) system, have hugely accelerated the functional genomic research in cancer. The first genome-wide genetic screen in human cells was performed in 2014 by lentiviral delivery of a genome-scale CRISPR-Cas9 knockout (GeCKO) library targeting more than 18,000 genes^274^ (Fig. 1). Genetic screens, by introducing perturbations into genes at a large-scale, represent an important tool to systematically classify the human genetic elements into functional groups and biological processes, in particular for metabolic pathways (Fig. 6). Compared to traditional short hairpin RNA (shRNA)-based system for performing lethality screens, CRISPR/Cas9 loss-of-function libraries provide a much greater screening sensitivity, since incomplete knockdown by shRNA sometimes does not produce loss-of-function phenotypes. Moreover, CRISPR/Cas9 library screen displays less variation in the data, less off-target effects thus resulting in a low False-discovery rate (FDR) and better consistency across cell lines.^275,276^ CRISPR/Cas9 screens have been able to identify genes essential for OXPHOS,^277,278^ for redox homeostasis,^279^ ferroptosis^280^ and cell fitness.^281–283^ CRISPR/Cas9 screening and MFA, allow the study of dispensability and interactions between set of genes encoding enzymes leading to the identification of key nodes within glycolysis and PPP.^284^ By performing CRISPR/Cas9 screen in Human plasma-like medium (HPLM) compared to traditional media, a massive effect of medium composition on gene essentiality in human cells has been described. Entire sets of essential genes vary with natural cell-intrinsic heterogeneity, suggesting that future genetic screens in HPLM will define new targetable liabilities across diverse human cancers.^285^ The advent of CRISPR screening technology now provides a rapid, high-throughput means to perform functional characterization of large gene sets. The functional relationships between key enzymes, transcription factors and transporters allowed the identification of multiple interactions within and across metabolic pathways. More importantly, its wide application in concert with the metabolome analysis allowed the annotation of many essential metabolic genes in human tumours and the identification of context-dependent dispensability that can be therapeutically targeted.

## Metabolomics and bioinformatic approaches in precision medicine

High-throughput approaches such as metabolomics involve, even for the simplest assay, the processing of a huge quantity of data, which must be manipulated and mined to get to the underlying biological information. As for the other omics, this need is addressed by the heavy use of computational algorithms. After metabolite detection through the appropriate analytical approach (discussed above), pre-processing of the raw signals is performed to generate the correct data format for subsequent data processing, in which data are normalized to minimize technical and system bias, and in untargeted approaches metabolites are identified thanks to the availability of spectral databases. Over the last two decades, even more advanced signal processing techniques for MS- and NMR-based metabolomics have been proposed, including software for peak detection and alignment (e.g. XCMS, MZmine2, Open-MS, MS-DIAL, eRah, ADAP-GC, BinBase), which enable spectral annotations and identifications from big and complex data.^286–295^ Statistical analysis, including univariate and multivariate analysis, is used to identify significantly expressed metabolites, which are then linked to a biological process by functional analysis performed with specialized tools that map metabolites to known biochemical pathways according to the information collected in public databases such as KEGG.^296^ Finally, metabolomics data may be integrated with other omics data (transcriptomics, proteomics, or the microbiome) to fully picture the complexity of a biological system.^297,298^ Thus, computational methods are involved in metabolomics at least at three levels: primary data production and conversion into standardized formats, bioinformatic analysis of data collections for prediction or classification, and integrative analysis of metabolomics with other omics data. While the first level is heavily linked to the specific technology used for data production, the second and the latter approaches are applicable within a more general analysis framework and have been increasingly employed to the field of precision medicine. Bioinformatics is the perfect tool for precision medicine since it enables the integration of the omics biomarkers recognized in individuals or cohorts of patients. According to the guidelines established by the US National Research Council, multilayered molecular or omics data should be deposited in the Information Commons, which is an easily accessible repository containing also all the clinical and epidemiological information, when available, enabling a complete analysis of the linkages between the data layers, thus generating a network of knowledge. This network permits a categorization of diseases in taxonomic ranks, thus allowing precise diagnosis and therapy design.^299^ Data processing and statistical analysis for metabolomics have been extensively reviewed^300–302^ and are not the scope of this manuscript. Like for several other high-throughput disciplines, these tools are quite mature, while the field of integrative and artificial intelligence-based analysis of multiple datasets still poses significant challenges,^303^ including the harmonization of data from different metabolomic platforms and their combination with data from other high throughput technologies.

Historically, genomics was the first high-throughput discipline in which bioinformatic analysis was used to stratify patients.^304^ Over the years, using data generated with different omics platforms, there was the development of molecular classifiers capable of discriminating between samples derived from patients and normal individuals thus allowing patient stratification. Nonetheless, all the studies tackling the development of classifiers face the problem of multiple comparisons, large number of parameters determined on a (relatively) small number of cases, and the need for extensive validation to avoid overfitting issues. Despite these challenges, studies producing molecular classifiers are appealing to researcher in the oncology field due to the need to subset patients’ cohorts in potential responders and non-responders to specific treatments.^305^ To overcome the technical issues related to molecular classifiers, integrative analysis of multiomics data and spatially and temporally dynamic sampling of tumours have been suggested and attempted.^306,307^

The vast majority of approaches to integrative multiomics data analysis relies heavily on machine learning. In the recent years, there has been a lot of hype on the use of Artificial intelligence (AI) for multilayered data analysis, which has somehow obscured the notion that machine learning, an AI implementation, has been around for several decades and have been at the core of omics data analysis from the beginning. Certainly, the huge amount of information produced by the “big data era” has pushed the application of these computational techniques even further. Machine learning has been classified primarily into supervised and unsupervised; the first one requires labelled data (training data) to allow the model to learn the “known” patterns underlying the labelled data and then discriminate these patterns in data not yet seen (test data); conversely, the second type of learning does not require labelled data and its goal is to distinguish the underlying unknown patterns from large datasets in an unbiased manner. The most employed supervised learning tools are neural networks, support vector machines, decision trees, random forests, and hidden Markov models. Classic examples of unsupervised methods are clustering algorithms, such as hierarchical agglomerative clustering, k-means clustering, Principal component analysis (PCA), self-organizing map, and non-negative matrix factorization. Machine learning has been largely used in precision medicine, and it is now the main tool employed at the core of “deep data integration” (i.e. integration of datasets from exploration of interdependent systems, like gene expression and transcription factor binding data)^308^ and “broad data integration” (i.e. integration of several omics and clinical data to derive high-level properties or subclassifications of a disease, like cancer).^309^ While computational algorithms are already known, one of the major challenges in their application is the generation of a multidisciplinary attitude between physicians, biologists and bioinformaticians. As outlined by Duerr-Specht et al., in order to improve precision medicine, it is now mandatory not only to improve the organization and the technologies in informatics and data management, but it is also necessary (in a high sense) to focus on training of researchers.^303^

Indeed, the issue of combining multiomics data is more linked to devising a proper strategy than to developing novel analytical tools. Association analysis and classifier development, in fact, have already been successfully employed to metabolomics data like it was previously done on genomic, transcriptomic, or proteomic data to discriminate tumour subtypes.^310,311^ Similar to genome-wide association studies, metabolomic analysis have been performed on biofluids or tissue with the aim of identifying markers of susceptibility to different diseases, including breast, prostate, lung, colorectal, ovarian, and pancreatic cancer.^151,156,312–315^ These studies, however, are restricted to a single dimension of the sample’s biology, require validation on independent cohorts and can only find associations between patterns and a particular physiopathological state, which is not proof of a cause-effect nexus. This is usually provided by searching for specific biomarkers previously identified by the association analysis in disease models, as exemplified by the work of *Sreekumar* et al. on human and mouse prostate cancer.^146^ As genomics, transcriptomics and proteomics, metabolomics can be targeted or untargeted, and applied on biofluids, tissues, cell lines or model organisms. This gives the chance to integrate different layers of data from each omics platform, and to move from association studies to the identification of causal relationship and complex interrelations between the different levels of regulation of gene expression and metabolism – and their dysregulation—in cancer. As a simple example, data from genetic profiling and subtyping derived from gene expression analysis can be employed to perform supervised machine learning and to cross-validate metabolic data. Deep data integration is used to combine datasets underlying a common regulatory network (e.g. gene expression, metabolic, and transcription factor data), whilst broad data integration can be performed by parallel integration of multiomics and clinical data.^306,308,309^ These approaches allow to step forward from single level biology to systems biology, revealing interactions between the different layers of data that can be further exploited in disease models and validated in prospective cohorts. To avoid the generation of misleading data, it is critical to deeply characterize the disease models; as an example of this caveat, a recent meta-analysis demonstrated that most of the studies using JCA-1 cell line referred to it as a prostate cancer cell line, while it was actually derived from a bladder carcinoma.^316^ The same caution must be applied to avoid strain variability in both cell lines and animal models,^317^ and in the harmonization of data/sample collection from patients cohorts and tissue and biofluids biobanks.

## Conclusion and prospect

The intrinsically dynamic nature of cancer metabolism requires an equally dynamic research attitude.^318^ The recent technological and conceptual advances discussed in this review, from metabolomics to single-cell approaches, have significantly broaden the research scenario making cancer metabolism one of the most vibrant and prolific area of cancer biology research.^3^ Despite being the youngest and less employed omics technique, metabolomics is the technology that has mostly driven this change and it has demonstrated an enormous potential to influence the future of cancer research with concrete clinical applications in oncology. In particular, the analysis of biological fluids, which have an easy access and require minimal sample processing, is what is closest to be translated into a clinical setting and it is already widely employed for biomarker discovery, diagnosis, identification of new drug targets and for clinical trials monitoring.^319,320^ However, these analyses still require a deeper comprehension of how the read outs and the quantifications depict a realistic picture of the human physiology and to what extent a metabolite profile in biofluids reflects the metabolic milieu of the tumour. Moreover, metabolomic analyses and the underlying data processing present several challenges in the standardization and often there is not a unique approach, but each study is context-specific thus researchers should have deep multidisciplinary and computational education.^321^ Besides metabolomics, over the last 40 years, the development, the increased accessibility, power and resolution of new technologies have pushed cancer research as never before. In particular, the advent of high-throughput and high-content single-cell technologies provided unprecedented tools to investigate cancer biology at cellular resolution (Fig. 1). The upgrade from bulk to single-cell analysis enabled the researchers to face the pressing challenge of elucidating the complexity of heterogeneous diseases, like cancer, and the underlying molecular pathways driving them thus describing the cancer framework in depth with both a quantitative and qualitative analysis. Caution must be still taken because none of the above-mentioned technologies, if employed alone, can completely analyse the metabolic status of a tissue. The choice of the most appropriate technique to extricate oneself from a complex biological system, such as TME, must always balance between sensitivity and resolution, cost and feasibility (Table 2). These metabolic tools should be employed in a complementary way, merging descriptive research to a functional study, thus paving the way to innovative approaches to research, diagnosis, and therapies in cancer. By combining these techniques—and thus exploiting several levels of orthogonality—we will be able to expand our understanding of the tumour and pave the way for new anti-cancer strategies. The biggest challenge, which everyone working in field is currently facing, is the lack of interlaboratory harmonization strategies for analytical procedures, which must be one of the next goals in the field in order to implement the computational tools for the development of open-source databases and ultimately advance metabolic studies in cancer research.

**Table 2:** Pros, cons and sample types of the main metabolomic technologies for the analysis of cancer metabolism

Method	Pros	Cons	Sample types
Gas chromatography-Mass spectrometry (GC-MS)	• High sensitivity for volatile metabolites • High-resolution separation • Analysis of different groups of metabolites simultaneously • Large linear range	• Long sample preparation (derivatization step for non-volatile metabolites) • Thermolabile compounds cannot be analysed • Slow dynamic range speed • Slow analysis	• Cultured cells • Supernatant • Biofluids • Tissues • Organoids
Liquid chromatography-Mass spectrometry (LC-MS)	• Simple and fast sample preparation (derivatization not usually required) • Wide coverage of metabolites • Thermolabile compounds can be analysed • High sensitivity • Soft ionization	• Ion suppression • Expensive • Slow analysis	• Cultured cells • Supernatant • Biofluids • Tissues • Organoids
Capillary electrophoresis-Mass spectrometry (CE-MS)	• Low sample volume • High resolution • Rapid analysis • No derivatization required	• Affected by salt • Low stability compared to GM- and LC-MS • Poor reproducibility and sensitivity	• Cultured cells • Supernatant • Biofluids • Tissues • Organoids
Direct infusion-Mass spectrometry (DI-MS)	• High-throughput • Simple data processing	• Do not distinguish the isomers	• Supernatant • Biofluids
Matrix-assisted laser desorption ionization-Mass spectrometry (MALDI-MS)	• Low sample volume • Fast analysis • High tolerance towards salts • Suitable for high MW metabolites • Non-destructive	• Low reproducibility • Hard identification due to complex matrix	• Cultured cells • Supernatant • Biofluids • Tissues • Organoids
Mass spectrometry imaging (MSI)	• In situ detection • Preserve histological integrity	• High resolution is time-consuming • No functional profile	• Cultured cells • Tissues • Organoids
Direct real-time analysis (DART)	• No sample processing • Direct analysis	• Not suitable for polar compounds	• Supernatant • Biofluids
Nuclear magnetic resonance (NMR)	• No separation • Structural information • High reproducibility • Fast sample preparation • Non-destructive	• Low sensitivity • Expensive instrument • Some chemical classes are not detected	• Cultured cells • Supernatant • Biofluids • Tissues • Organoids
Metabolic flux analysis (MFA)	• Quantitative analysis and information on metabolites fate	• Isotope tracing is expensive • Compartment specific flux	• Cultured cells • Tissues • Organoids
Extracellular flux analysis (EFA)	• Real time measurement • High feasibility • Relatively cheap	• Bulk analysis • Only relative and indirect measurement • Cell purification is required	• Cultured cells • Organoids
Single-cell RNA-sequencing (scRNAseq)	• Low cell number • High-resolution • Unbiased gene expression analysis • Metabolic phenotype at mRNA level	• Expensive • Temporal discordance between mRNA and protein/functional effect • Do not consider post-transcriptional and post-translational mechanisms	• Cultured cells • Tissues • Organoids
Single-cell metabolomics (SCM)	• Low cell number • High resolution • High-throughput	• Challenge of combining single cells sorting and metabolism quenching • Need of high sensitivity and throughput analytical platform	• Cultured cells • Tissues • Organoids
Single-cell energetic metabolism by profiling translation inhibition (SCENITH)	• Functional analysis coupled to large phenotype • Fast and simple sample preparation and analysis	• Only relative and indirect measurement • Not suitable for cells with undetectable level of protein synthesis	• Cultured cells • Tissues • Organoids
Cytometry by Time-of-flight (CyTOF)	• High-dimensional • High-throughput • Metabolic phenotype at protein level	• Not suitable for weakly expressed markers • Requires advanced biostatistics and bioinformatics	• Cultured cells • Tissues • Organoids
Met-flow	• Fast and single-cell analysis • Metabolic phenotype at protein level	• Measurements are indirect • No functional profile	• Cultured cells • Tissues • Organoids
In situ dehydrogenase activity assay	• Single-cell analysis in native microenvironment • Functional profile	• Measurement at saturated substrate concentrations	• Cultured cells • Tissues • Organoids
Genetic screening	• Precise gene targeting (few off-targets) • Robust signal derived by permanent gene disruption	• Complicated to perform • For some types of studies, it is not good having a permanent gene disruption	• Cultured cells • Organoids
